# Southeast Asia initiative to combat SARS-CoV-2 variants (SEACOVARIANTS) consortium

**DOI:** 10.12688/wellcomeopenres.20742.1

**Published:** 2024-04-11

**Authors:** Le Nguyen Truc Nhu, Mary Chambers, Narisara Chantratita, Phaik Yeong Cheah, Nicholas P.J. Day, Wanwisa Dejnirattisai, Susanna J. Dunachie, Alba Grifoni, Raph L. Hamers, Jennifer Hill, E. Yvonne Jones, Paul Klenerman, Juthathip Mongkolsapaya, Gavin Screaton, Alessandro Sette, David I. Stuart, Chee Wah Tan, Guy Thwaites, Vu Duy Thanh, Lin-Fa Wang, Le Van Tan, SEACOVARIANTS Consortium

**Affiliations:** 1 Oxford University Clinical Research Unit, Ho Chi Minh city, Vietnam; 2 Centre for Tropical Medicine and Global Health, Nuffield Department of Medicine, University of Oxford, Oxford, England, UK; 3 Department of Microbiology and Immunology, Faculty of Tropical Medicine, Mahidol University, Bangkok, Thailand; 4 Mahidol Oxford Tropical Medicine Research Unit, Faculty of Tropical Medicine, Mahidol University, Bangkok, Thailand; 5 Division of Emerging Infectious Disease, Research Department, Faculty of Medicine Siriraj Hospital, Mahidol University, Bangkok, Thailand; 6 Division of Structural Biology, Nuffield Department of Medicine, University of Oxford, Oxford, England, UK; 7 La Jolla Institute for Immunology, San Diego, California, USA; 8 Oxford University Clinical Research Unit Indonesia, Faculty of Medicine Universitas Indonesia, Jakarta, Indonesia; 9 Chinese Academy of Medical Science (CAMS) Oxford Institute (COI), University of Oxford, Oxford, England, UK; 10 Wellcome Centre for Human Genetics, Nuffield Department of Medicine, University of Oxford, Oxford, England, UK; 11 Programme in Emerging Infectious Diseases, Duke-NUS Medical School, Singapore, Singapore

**Keywords:** SARS-CoV-2 variants, pandemic responses

## Abstract

A strong and effective COVID-19 and future pandemic responses rely on global efforts to carry out surveillance of infections and emerging SARS-CoV-2 variants and to act accordingly in real time. Many countries in Southeast Asia lack capacity to determine the potential threat of new variants, or other emerging infections. Funded by Wellcome, the Southeast Asia initiative to combat SARS-CoV-2 variants (SEACOVARIANTS) consortium aims to develop and apply a multidisciplinary research platform in Southeast Asia (SEA) for rapid assessment of the biological significance of SARS-CoV-2 variants, thereby informing coordinated local, regional and global responses to the COVID-19 pandemic. Our proposal is delivered by the Vietnam and Thailand Wellcome Africa Asia Programmes, bringing together a multidisciplinary team in Indonesia, Thailand and Vietnam with partners in Singapore, the UK and the USA. Herein we outline five work packages to deliver strengthened regional scientific capacity that can be rapidly deployed for future outbreak responses.

## Plain language summary

Our project strengthens local scientific capacity in South East Asia (SEA) and therefore enables the rapid assessment of SARS-CoV-2 variants as they emerge within the region. While COVID-19 remains a global pandemic, future emerging infections caused by a novel virus is an inevitable event, with SEA being a global hot-spot for pathogen emergence. Consequently, the research capacity built, the scientists trained and the research network formed as part of this project will lay the foundation for future locally-led outbreak responses. Our project will demonstrate that novel research platforms can be set up in other low and middle income countries to address the unprecedented challenges presented by emerging infections.

## Disclaimer

The views expressed in this article are those of the authors. Publication in Wellcome Open Research does not imply endorsement by Wellcome.

## Introduction

### Background

Tracking the evolution of new variants of SARS-CoV-2 and understanding their impact on disease phenotype has been one of the major challenges of the COVID-19 pandemic ^1^. With SARS-CoV-2 continuing to circulate around the world, new variants with structural changes in the spike protein that can evade existing infection or vaccine-derived immunity will almost certainly continue to emerge. The rapid assessment of the impact of new SARS-CoV-2 variants on existing population immunity induced by vaccination and/or infection ^2– 5^ and their clinical consequences of infection is critical to public health responses. However, there is limited capacity in the low- and middle-income countries (LMICs) of Southeast Asia (SEA) to make such assessments. Building on existing expertise and laboratory capacities within the Wellcome Africa Asia Programmes (AAPs) situated in Thailand, Vietnam and Indonesia, we are developing a new platform that will enable the rapid biological assessment of SARS-CoV-2 variants within SEA. Our platform utilises a multi-disciplinary research approach, encompassing structural biology, and antibody and T cell response analyses to generate timely data on the potential threat of new SARS-CoV-2 variants. These research activities are supported by policy-maker and public engagement components that will bring research results into practice, and will utilize engagement activities to inform the design of laboratory-based research.

Structural biology is integrated into SARS-CoV-2 genomic surveillance within the UK and has been successfully used to accurately predict impacts of variants of concern (VOCs) on vaccines and therapeutics ^3^. By integrating all known antibody binding/therapeutic target data with structural insight, it is possible to identify mutations/residues most likely to have a significant escape impact, informing subsequent *in vitro* assessment. Coupling structural biology analysis with laboratory assays will help generate timely data on the potential biological significance of SARS-CoV-2 variants emerging in SEA relevant to local settings, key to supporting policy makers with evidence-based decisions.

Although neutralising antibodies correlate with protection against COVID-19 ^6– 9^, T-cells are a vital component of antiviral defence, especially against severe disease. For example, the Omicron variant evades pre-existing antibody neutralisation ^3^ but T-cell responses are preserved in 70-80% ^4, 10– 15^, likely playing a major role in the low rates of hospitalization and death in vaccinated populations and contrasting with Omicron’s high mortality in under-vaccinated people ^16^.

Several countries in SEA border mainland China, where SARS-CoV-1 (responsible for the 2003 outbreak) ^17^ and SARS-CoV-2 first emerged ^18^. In this region, numerous SARS-CoV-1/2 related viruses of the subgenus *Sarbecovirus* have been discovered in a wide range of animal species, including bats and pangolins ^19– 23^. In rural SEA, people are regularly in close contacts with animals ^24^. Therefore, these populations may have a different immune landscape compared to those in other geographic regions due to differences in past pathogen exposure ^25^ and/or human leukocyte antigens (HLA) repertoire. Therefore, findings from high-income countries, currently dominating the scientific literature, might not translate into the SEA setting.

Besides pathogen exposure and population genetics, vaccination is a major factor generating regional differences in immune landscape. High-income countries have predominantly deployed mRNA and viral vector vaccines. In SEA, more diverse vaccine products have been used, including mRNA (BNT162b2 and mRNA-1273), adenoviral vector (Oxford-AstraZeneca), whole-inactivated virus (Sinovac, SinoPharm) and protein subunit (Abdala, from Cuba) vaccines ^26– 28^. Relatively little is known about the immunogenicity of some of these vaccine candidates (e.g., Abdala ^29^), and the T-cell responses to whole-killed virus vaccines ^30^. The heterogeneity in vaccine products used, coupled with the potential differences in the pre-existing immune landscape and diverse HLA repertoires, make SEA a unique setting for studies characterising immune responses and clinical consequences of infection with VOCs.

The Thailand and Vietnam AAPs have more than four decades’ experience of engaging with the public and policy makers. The scale of COVID-19 required an adjustment in priorities and new approaches to engagement. It has opened new partnerships and built a wider community of local engagement avenues. The AAPs have developed new models of on-line public engagement and training and identified those marginalised and made most vulnerable by the pandemic. On-line communications have strengthened collaborations and activities across SEA and form the basis for the new framework to be created by the SEACOVARIANTS consortium with funding from Wellcome to communicate and engage concerning virus variants and their threat to public health. In Vietnam, an Outbreak Advisory Board (OAB) was established with representatives from the Ministry of Health and major national and international institutes, including the WHO. The OAB provides a live interface between national policy makers and AAP researchers, enabling bidirectional flow of information. In SEACOVARIANTS we aim to extend the model to Indonesia to ensure our research findings translate rapidly into practice. In Indonesia, the AAP has advised and collaborated with MOH and local Health Offices to assist in laboratory diagnosis, genomic surveillance and advanced data analysis. The Thailand AAP facilitates and routinely consults various public and young persons’ advisory groups; such as the decade-old Tak Province Community Ethics Advisory Board founded ^31^; on their research and public health programmes including on COVID-19 research.

### Aims

Our overarching aim is to develop and apply a multidisciplinary research platform in SEA for rapid assessment of the biological significance of SARS-CoV-2 variants, thereby informing coordinated local, regional and global responses to the COVID-19 pandemic. Our specific aims are:

1. Establish a new SEA research platform that supports locally led investigations evaluating the biology of emerging SARS-CoV-2 variants.
2. Employ state-of-the art structural biology to provide rapid prediction of the ability of new variants to evade host immunity and drugs.
3. Evaluate the impact of VOCs on antibody and T-cell responses in SEA populations, and the clinical consequences of infection.
4. Create a framework for effective communication and engagement with policy makers and the public concerning new virus variants and their potential to threaten public health.

### Research approaches

**Figure 1.**
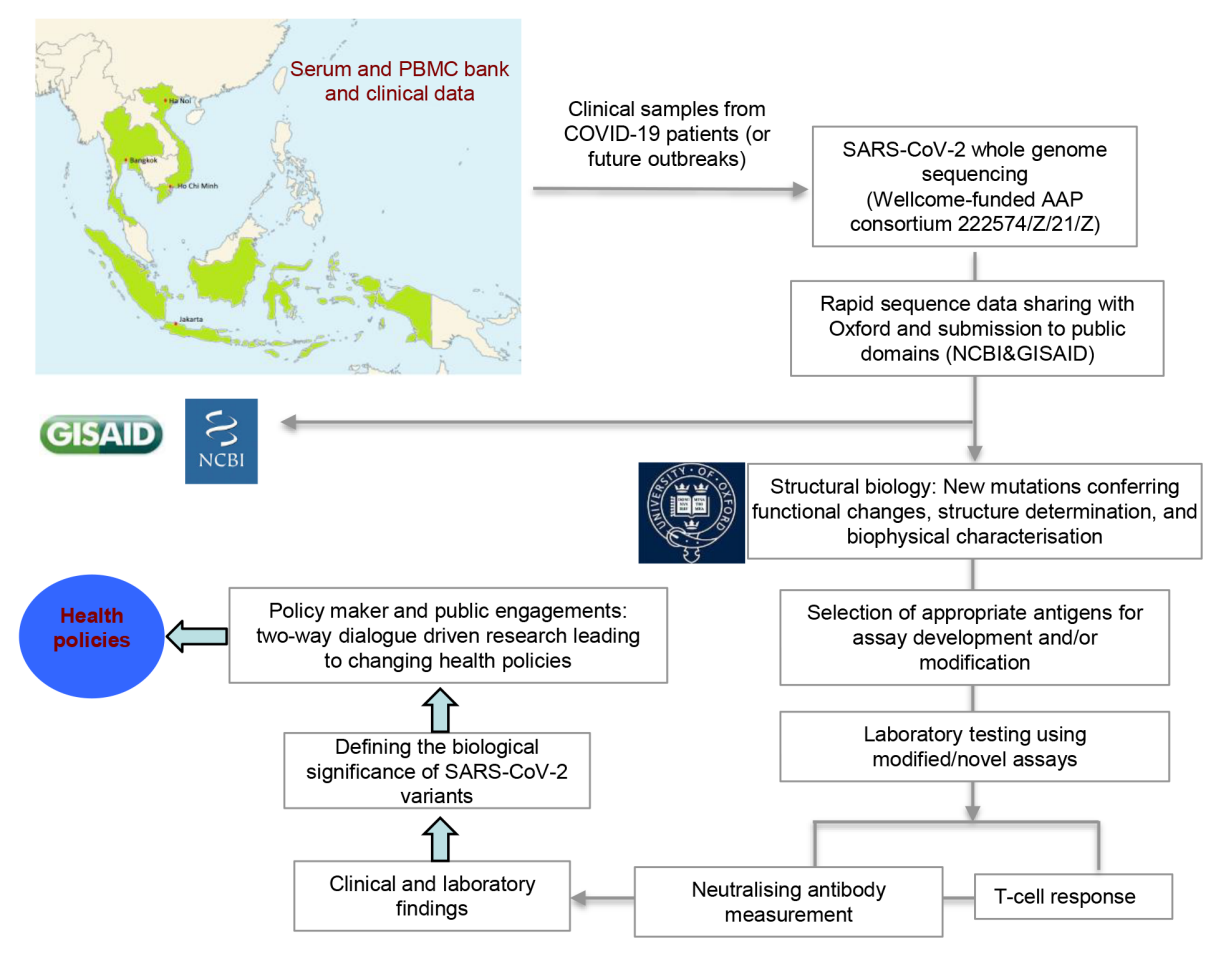
An outline of our approaches from genetic characterization of variants of concern to generation of immunological data to inform public health response. The platform that enables rapid biological assessment of SARS-CoV-2 variants within SEA is being built on existing expertise and laboratory capacities within Vietnam and Thailand Wellcome Africa Asia Programmes. This will allow a locally-led research response to the COVID-19 pandemic and future outbreaks.

**Figure 2.**
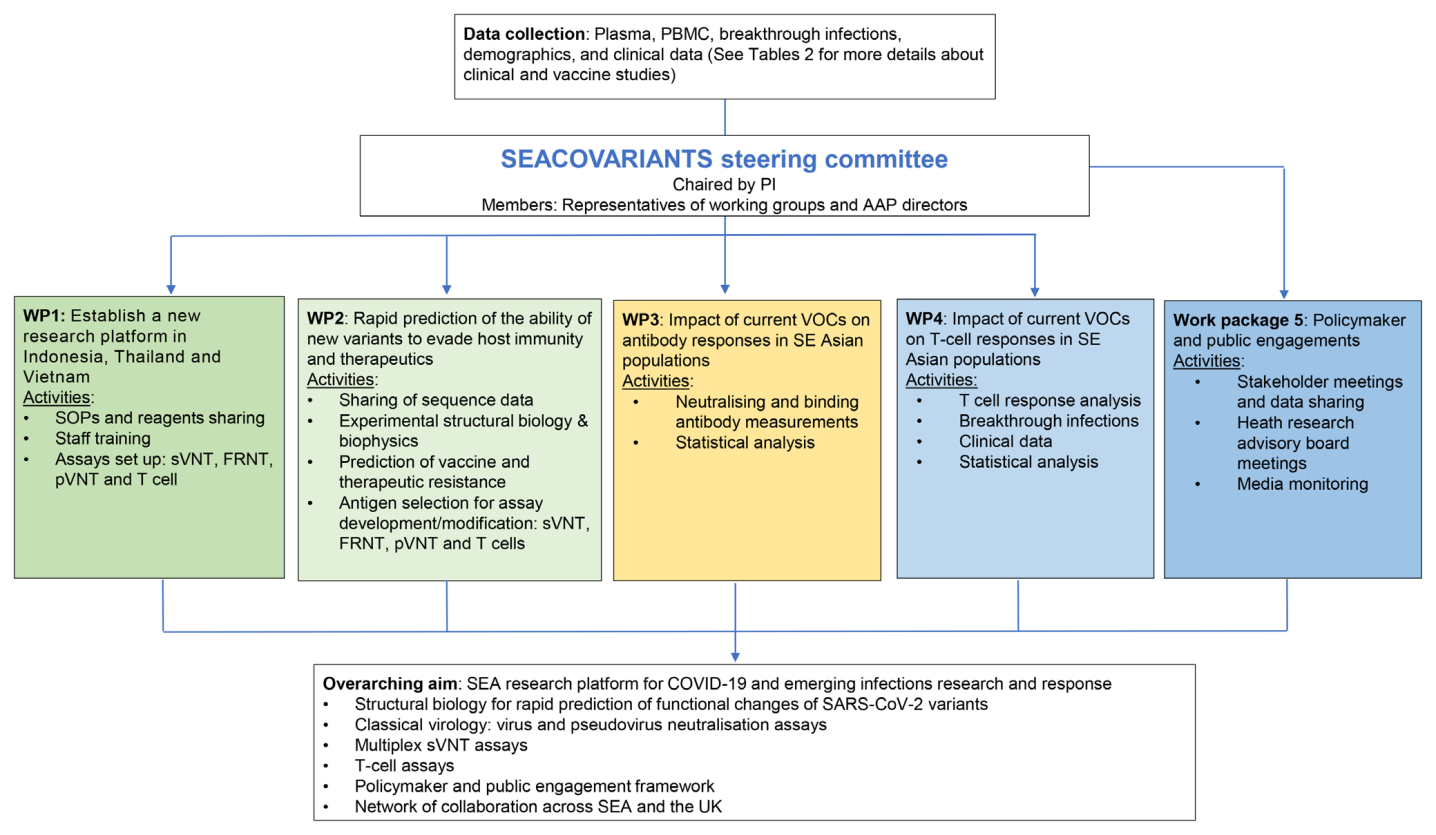
SEACOVARIANTS work packages. Our objectives are delivered through five Work Packages (WPs). The core laboratory research activities are described under WP3&4, supported by WP1, 2 & 5. WP1 will establish a foundation for the delivery of the laboratory-based analysis (WP3&4), while WP2 will inform key analysis undertaken under WP3 & 4. WP5 brings research results (WP3 & 4) into practice and utilizes engagement activities to inform the design of WP3 & 4.

Our approaches to data generation and public health impact are outlined in Figure 1. Our objectives are delivered through five work packages (WPs), with work under aim 3 subdivided into WP3 (antibody responses) and WP4 (T-cell responses) ( Figure 2). The core laboratory research activities are described under WP3&4, supported by WP1, 2 & 5. More specifically, WP1 will establish a foundation for the delivery of the laboratory-based analysis (WP3&4), while WP2 will inform key analysis undertaken under WP3 & 4. Finally, WP5 aims are to bring research results (WP3 & 4) into practice and to utilize engagement activities to inform the design of WP3 & 4.

***WP1 (objective 1)**: Establish a new research platform in Indonesia, Thailand and Vietnam*

Laboratory capacity development will be accomplished through sharing of SOPs and reagents, and training of local laboratory staff during lab visits, and co-supervision of PhD students. Table 1 illustrates existing and laboratory capacity in Indonesia, Thailand and Vietnam as well as capacity to be developed through the work of the consortium.

**Table 1.** Existing and required laboratory capacity in three research sites.

	BSL3 facility	N and S protein binding antibody	Multiplex sVNT	FRNT	Pseudovirus	Ex vivo IFN-γ ELISpot	Intracellular staining	CellTrace Violet proliferation
Indonesia	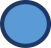	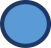	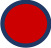	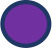	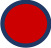	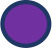	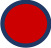	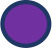
Thailand	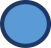	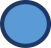	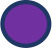	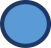	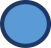	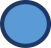	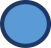	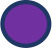
Vietnam	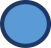	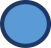	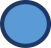	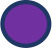	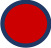	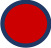	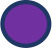	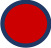

**Note to Table 1**: Blue circles: existing capacities for SARS-CoV-2, red circles: required capacities, purple circles: capacities available for other viral pathogens, and deployable for SARS-CoV-2. BSL = BioSafety Level, sVNT = surrogate Viral Neutralisation Test, FRNT = Focus Reduction Neutralisation Test, ELISpot = Enzyme-Linked Immunosorbent Spot.

***WP2 (objective 2):** Rapid prediction of the ability of new variants to evade host immunity and therapeutics*

Building on approaches implemented for UK surveillance structural analyses is used to assess the impact of mutations arising in SEA on responses from current vaccines and infection, as well as on therapeutics. These insights will inform the selection of appropriate antigens for pseudovirus, surrogate virus and focus reduction neutralization test (PVNT, sVNT and FRNT) and T-cell assay development/adjustment. Potentially significant mutations will be experimentally dissected by structural analysis (via cryo-EM or crystallography), pseudovirus analysis, and biophysical characterization of changes to binding to representative monoclonal antibodies, therapeutic antibodies and ACE2 receptor ^3^.

***WP3 (objective 3)**: Impact of current VOCs on antibody responses in SEA populations*

We focus on variants currently circulating in the region (including sublineages of Omicrons such as XBB.1.5 and XBB.1.16), and future VOCs arising during the timeframe of the award. Antibody responses will be assessed using a panel of sera collected from individuals with different infection and/or vaccination status ( Table 2).

**Table 2.** Available cohorts for analysis. Antibody responses will be assessed using a panel of sera collected from individuals with different infection and/or vaccination status from different study cohorts in Indonesia, Thailand and Vietnam.

Site	Cohort	Primary doses	Booster	Population
**Vietnam**	Vaccine	ChAdOx1-S	BNT162b2	HCW
		Abdala	Abdala or BNT162b2	General population
	SARS survivors	Pending	Pending	General population
	Natural Infection			General population
**Indonesia**	Vaccine cohort	CoronaVac	mRNA-1273	HCW, general population and pregnant women
	Natural Infection			General population
**Thailand**	Vaccine cohort	CoronaVac	B162b2 or ChAdOx1-S	General population
	Natural Infection			General population

Neutralising antibodies are measured using a combination of live virus focus reduction neutralisation tests, high throughput Luminex multiplex surrogate viral neutralisation tests (sVNT) and/or pseudovirus neutralisation tests (PVNT) ^3^. Although live virus neutralisation assays remain the gold standard, these assays are limited in throughput compared with alternatives, and require live viruses and BSL3 facilities. The Luminex sVNT accommodates >20 antigens (sufficient to cover all VOCs and a wide range of sarbecoviruses, including SARS-CoV-1) in one reaction tube and can be carried out at BSL2 ^32^. The FRNT, sVNT and PVNT assays generate complementary data and will allow a broad assessment of cross reactivity between VOCs and sarbecoviruses.

***WP4 (objective 3):** Impact of current VOCs on T-cell responses in SEA populations*

The impact of VOCs on T cell responses is assessed using three complementary assays: *ex vivo* interferon gamma (IFN-γ) ELISpot assay, intracellular staining (ICS), and the CellTrace™ Violet (CTV) proliferation assay ^33^. The ELISpot assay measures the effector T-cell response to peptides spanning SARS-CoV-2, and can be adapted to analyse T-cell responses to mutated regions of SARS-CoV-2 using custom-made peptide sets to look for T-cell escape ^34– 36^. The ELISpot protocol implemented has been optimized to be highly sensitive and specific ^33^ delivering consistent results across UK laboratories ^35– 37^. ICS gives key information about the character of the T-cell response, including the helper CD4 or cytotoxic CD8 composition and memory phenotype ^33, 35, 37^. The CTV proliferation assay measures the central memory CD4 and CD8 response ^33, 38^, and therefore can also identify cross-reactivity between VOCs and previous exposure with other sarbecoviruses, and/or common cold coronaviruses. There will be opportunity to evaluate the impact of baseline T-cell responses to the “common cold” coronaviruses (229E, NL63, OC43, HKU-1) on the T-cell response to ancestral strains and VOCs after vaccination through co-ordination with current research in Oxford. The platform will benefit from collaboration with La Jolla Institute for Immunology. La Jolla lab was able to rapidly support investigation of the T-cell response to Omicron by shipping peptides to five global labs within 2 weeks of release of viral sequence, and will collaborate to supply SARS-CoV-2 peptides and expertise for this study ^15, 39^.

The T-cell response to circulating VOCs will be compared between cohorts of individuals with COVID-19, between recipients of different primary vaccine courses and between populations ( Table 2), to define protection of populations against current and emerging VOCs.

Through clinical studies, we have been collecting meta clinical data during hospitalization and samples from COVID-19 patients admitted to our collaborating hospitals ^40, 41^. This has enabled us to start assessing the severity associated with respective SARS-CoV-2 variants, and will allow us to relate immune responses to individual variants with severity profiles.

***WP5 (objective 4):** Policymaker and public engagements*

SEACOVARIANTS will synthesizes data about immune/vaccine/therapeutic escape potential of SARS-CoV-2 variants allied with clinical data and provide critical information for policy makers as the pandemic progresses in SEA. Over decades, we have established strong connections with health policy makers (including Ministry of Health (MOH)) in our respective countries. We have been able to rapidly disseminate our COVID-19 research findings to in-country local policy makers using various approaches; 1) OAB meetings organized every quarter, 2) regular written reports, 3) oral presentations, 4) personal communication, 5) consultation meetings, and 6) press releases. In response to the Omicron emergence, Vietnam AAP Director Prof Guy Thwaites and lead consortium PI Tan were invited to attend informal consultation meetings with the Director of Health Service and the Party Committee Secretary of HCMC in November 2021. On 22 ^nd^ April 2022, lead applicant Tan presented findings from our on-going Wellcome-funded genomic surveillance project at an MOH meeting. In Indonesia, the team has catalysed high throughput genomics assays and monthly bulletins with recommendations for the MOH.

In many LMICs the public, and in particular young people, have little opportunity to contribute to public health decision making. Adults strongly influence the decisions made for children. SEACOVARIANTS is creating platforms for public involvement in this project, providing valuable feedback on public perceptions about SARS-CoV-2 variants. Youth-led engagement and adult involvement from the outset will inform researchers and stakeholders and support public health measures preventing and controlling new outbreaks. The partnership with young people through media and digital engagement will increase public awareness of the benefits of research.

Engagement activities are led by senior engagement practitioners imbedded in the AAPs with decades of experience in LMICs, including:

**Health Research Advisory Groups**: existing public adult groups in Vietnam and Thailand give feedback on study design, science findings and their wider implications. They will offer insights into wider public perceptions about variants, surveillance etc.

**Youth Working Groups**: local groups of young people aged 18–24 have been established through our existing youth networks. They are ambassadors for the project and bring youth voices to researchers and stakeholders around new variants, and collaborate with the research teams to develop and lead engagement activities relevant to their communities.

**Media monitoring**: Tracking misinformation and public concerns regarding VOCs in social media informs the content of engagement activities to be relevant to local populations. We have a successful track record of monitoring COVID-19 related media across SEA to inform social media and public health messaging content.

Public and youth engagement are raising the profile of the research, create opportunities for public voices in the process, build our understanding of public priorities regarding pandemics, and support the case when research is presented to policymakers. The partnerships and engagement models developed in each site will build capacity for ongoing engagement for the researchers, and within the research centres.

### Collaborative approach

From many years of collaborative experience, from our perspective a key lesson learnt is that export of pathogen and human samples to high-income countries for analysis can be slow or even impossible. Pandemic and outbreak investigation require rapid access to sophisticated laboratory techniques. Our platform is therefore designed to enable the bulk of the work to be completed locally, by establishing a network of support between labs, including in Oxford. Experimental structural biology, biophysics and monoclonal antibody testing will be performed in Oxford due to the need for specialist infrastructure (e.g., synchrotron radiation source, cryoEM), however, provision of tools to visualise, and expertise to assess, the likely impact of mutations will be developed, to underpin informed decision making across all partners.

Allocation of resources and training is based on the need from each project partner to ensure all participating sites have sufficient resources and capacity to deliver the project at the highest standard. The data will be made available at the time of publication. Contributing individuals are acknowledged and/or named as authors in scientific publications, according to the authorship criteria.

The project is overseen by the consortium Steering Committee, chaired by the lead applicant with members including co-investigators, public health stakeholders in respective countries and AAP directors ( Figure 2). The committee meets virtually at least every six months to discuss the overall strategic direction of the project and progress. Additionally, working groups ensure specific WPs are delivered as scheduled.

## Conclusion

Emerging viruses pose a significant threat to healthcare systems worldwide, as exemplified by the COVID-19 pandemic. Yet, another new pathogen, causing ‘disease X’, will almost certainly emerge within the next decades. Pandemic preparedness is one of the top priorities of the WHO. Asia is home to more than 50% of the world’s population. The population in Indonesia, Thailand and Vietnam combined accounts for over 65% of the >655 million people living in SEA. Fragile health systems, dense populations with people and animals living close together, rapid urbanization and economic development, yet with stark health inequalities make the region highly susceptible to emerging pathogens ^42^.

A critical component of pandemic preparedness and response is to establish essential capacities that enable rapid and robust scientific laboratory, epidemiological, clinical and social research within the most relevant settings and structures. Alongside the development of laboratory capacity in the region, the SEACOVARIANTS consortium will graduate a cohort of local scientists, including post-doctoral researchers, PhDs and research assistants with expertise in structural analysis, classical virology, advanced T-cell immunity and serology in SEA. The project helps strengthen our longstanding networks with policy makers and key stakeholders in respective countries, bring our research into practice, and further establish a scientific relationship between SEA and the UK. Collectively, the SEACOVARIANTS will provide proof-of-principle that such advanced laboratory tools can be applied effectively in LMICs and thereby help SEA and the world to better prepare for future pandemics.

## Data Availability

No data are associated with this article.
