# Supplementary material for: Southeast Asia initiative to combat SARS-CoV-2 variants (SEACOVARIANTS) consortium
Source: Wellcome Open Res. 2024 Apr 11;9:181. [Version 1] doi: 10.12688/wellcomeopenres.20742.1 (PMC11252647; doi:10.12688/wellcomeopenres.20742.1)
Supplement: Peer review history

# Reviewer report: Reviewer response for version 1

**DOI:** 10.21956/wellcomeopenres.22953.r87710

**Published:** 2024-07-16

**Authors:** Takamasa Ueno, Isaac Ngare

**Affiliations:** 1 Kumamoto University, Kumamoto, Japan; 2 Joint Research Center for Human Retrovirus Infection, Kumamoto University, Kumamoto, Kumamoto Prefecture, Japan

The original version of this report was made by a PhD student (currently under evaluation for PhD), Isaac Ngare in my lab, and then I edited and approved it.

 Le Nguyen Truc Nhu et al, provide a detailed plan of the Wellcome-funded southeast Asia initiative to combat SARS-CoV-2 variants (SEACOVARIANTS) activities in the southeast Asia region where SARS-CoV-2 surveillance is underdeveloped and the majority of rural populations are in close contact with animals making them susceptible to zoonotic spillovers. The manuscript is clearly written and provides a detailed and elaborate step-by-step account of the consortium’s plan to combat the emergence of new SARS-CoV-2 variants of concern. There are however a few concerns that need to be addressed to improve the clarity of the manuscript.

 1. The tone of the manuscript appears to be set in the middle of the COVID-19 pandemic while in many parts of the world, COVID-19 has been downgraded to the public health threat level of seasonal flus. This distinction is especially important because it has implications in the testing and detection of COVID-19 infections in public hospitals. As of 2024, many countries in Africa, and maybe high income countries, had stopped mass testing for COVID-19 unless requested by the client at their own cost. How is the situation SEA currently? If there are no government/insurance-funded tests, will this initiative carter the costs of COVID-19 tests for all the cases presenting with flu-like symptoms before sampling the positive cases? I believe this may have significant cost implications and the authors could clarify it.

 2. Another concern is how the findings from antibody and T cell responses will be converted to policy. Although antibody and T cell response are correlates of protection, the threshold below which protection is lost has been elusive and could vary across assays in WP3 and WP4. If for instance responses against a new variant are 2-fold lower than the prevalent variant, is this difference biologically significant to justify changes in policy? Also, the magnitude of antibody and T cell responses to new variants will significantly be influenced by the duration since vaccination or exposure to preceding variants. A clarification on how these factors will accounted for before inclusion in policy could be informative to the reader.

Does the article adequately reference differing views and opinions?

Yes

Are all factual statements correct, and are statements and arguments made adequately supported by citations?

Yes

Is the Open Letter written in accessible language?

Yes

Where applicable, are recommendations and next steps explained clearly for others to follow?

Yes

Is the rationale for the Open Letter provided in sufficient detail?

Yes

Reviewer Expertise:

immunology, virology, HIV/AIDS, SARS-CoV-2, a member of Gene-to-Phenotypes in Japan for COVID-19

We confirm that we have read this submission and believe that we have an appropriate level of expertise to confirm that it is of an acceptable scientific standard.

# Reviewer report: Reviewer response for version 1

**DOI:** 10.21956/wellcomeopenres.22953.r81748

**Published:** 2024-06-26

**Authors:** Pushpamali De Silva

**Affiliations:** 1 Harvard Medical School,, Boston, MA, USA

The SEACOVARIANTS initiative outlines a comprehensive multidisciplinary research platform aimed at rapidly assessing the biological significance of SARS-CoV-2 variants in SEA. The overarching goal is to inform coordinated local, regional, and global responses to the COVID-19 pandemic. This manuscript reports the clarity, feasibility, and potential impact of the aims, research approaches, and collaborative strategies proposed by SEACOVARIANTS.

**Strengths:**

- The aims of SEACOVARIANTS are well-defined and aligned with addressing critical gaps in understanding SARS-CoV-2 variants. Each aim contributes uniquely to the overall objective of enhancing pandemic preparedness through local capacity building, structural biology predictions, immune response evaluations, and effective communication strategies.
- The initiative employs a structured approach with five work packages (WPs) that integrate laboratory-based investigations (WP3&4) with foundational activities (WP1), structural biology predictions (WP2), and translation into policy and practice (WP5). This structured approach ensures robust data generation and impactful public health outcomes.
- A commendable aspect of SEACOVARIANTS is its emphasis on establishing and enhancing local research capacities across Indonesia, Thailand, and Vietnam. This includes laboratory infrastructure development, training of local personnel, and collaborative efforts with international partners to ensure sustainability beyond the project duration.
- The initiative leverages expertise across structural biology, virology, immunology, and public health, facilitated through a collaborative framework involving academic institutions, public health stakeholders, and policy makers. This approach fosters knowledge exchange and strengthens scientific relationships between SEA and the UK.
- SEACOVARIANTS incorporates robust public engagement strategies aimed at informing and involving policy makers and the public about virus variants and associated public health risks. These efforts are critical for fostering trust, addressing misinformation, and enhancing community participation in pandemic response efforts.

**Areas for Consideration:**

- While the initiative outlines ambitious goals, there should be careful consideration of operational challenges such as logistical coordination across multiple countries, ensuring uniformity in data collection protocols, and navigating regulatory landscapes for research involving human subjects and pathogens.
- Sustainability beyond the project period is crucial. SEACOVARIANTS should outline plans for continued funding, institutional support, and capacity building to ensure that local research capacities are maintained and expanded beyond the immediate scope of the initiative.
- While the initiative primarily focuses on biological and clinical aspects, integrating social sciences could provide deeper insights into community perceptions, vaccine acceptance, and behavioral responses to variants. This holistic approach would enhance the effectiveness of public health interventions.
- Given the global significance of variant surveillance, ensuring timely data sharing and accessibility is essential. SEACOVARIANTS should establish clear protocols for data management, sharing agreements, and open-access publication of findings to facilitate broader scientific collaboration and policy impact.
- Incorporating robust mechanisms for monitoring and evaluating the impact of SEACOVARIANTS on local health systems, policy development, and public awareness is vital. This will enable continuous improvement and evidence-based advocacy for sustaining and expanding similar initiatives.

Overall, SEACOVARIANTS represents a commendable collaborative effort that aligns with global health priorities and demonstrates a proactive approach to pandemic preparedness in the region.

Does the article adequately reference differing views and opinions?

Yes

Are all factual statements correct, and are statements and arguments made adequately supported by citations?

Partly

Is the Open Letter written in accessible language?

Yes

Where applicable, are recommendations and next steps explained clearly for others to follow?

Yes

Is the rationale for the Open Letter provided in sufficient detail?

Yes

Reviewer Expertise:

Immunology, Biology

I confirm that I have read this submission and believe that I have an appropriate level of expertise to confirm that it is of an acceptable scientific standard.

# Reviewer report: Reviewer response for version 1

**DOI:** 10.21956/wellcomeopenres.22953.r81753

**Published:** 2024-05-29

**Authors:** Sonika Bhatnagar, Shubhangi Gupta

**Affiliations:** 1 Netaji Subhas University of Technology, Dwarka, New Delhi,, India; 2 Computational and Structural Biology laboratory, Department of Biological Sciences and Engineering, Netaji Subhas University of Technology, New Delhi, Delhi, India

In the article, the authors have discussed about the Southeast Asia initiative to combat SARS-CoV-2 variants (SEACOVARIANTS) consortium funded by Wellcome. This represents a well-planned effort to develop a multidisciplinary research platform in Southeast Asia for the rapid assessment of the biological significance of SARS-CoV-2 variants. Authors have emphasized on the importance of pandemic preparedness and the need for developing robust laboratory, epidemiological, clinical, and social research  infrastructure in Southeast Asia.

 The consortium aims to strengthen local scientific capacity in Southeast Asia to enable the fast evaluation of emerging SARS-CoV-2 variants that could pose a potential threat to public health. The project seeks to build research capacity, train scientists, and establish a research network to lay the foundation for locally-led outbreak responses. This initiative would further lead to establishment of a new research platform, rapid prediction of the ability of new variants to evade host immunity, evaluation of the impact of variants on antibody and T-cell responses, as well as the creation of a framework for effective communication and engagement with the public and policymakers.

 The research approaches presented in the form of work packages are detailed, including the structural biology analysis of the potential SARS-CoV-2 variants, their assessment of antibody and T-cell responses along with engagement activities with policymakers and the public.

 Comments:

1. I am particularly impressed with the arguments in favor of starting the SEA SARS-CoV-2 consortium, including population density, close contact with animals, diversity of vaccine strategies, etc. The biology and thus the clinical profile of new and emerging variants is going to keep changing – and will need to be investigated. Therefore, apart from genomics, basic biology correlated with clinical findings will be key to monitoring the effects of new SARS-CoV-2 variants. This will also have considerable impact on capacity building and pandemic preparedness for the SEA countries. I am also happy with the plans for public engagement, particularly the youth, who can particularly help with contact within the community. This research could also help further evaluation of the differences in immune responses among individuals with different infections/vaccination status/HLA repertoires.
2. The authors state that “With SARS-CoV-2 continuing to circulate around the world, new variants with structural changes in the spike protein that can evade existing infection or vaccine-derived immunity will almost certainly continue to emerge.” It is my understanding that such variants that evade infection/vaccine derived immunity may emerge due to a large number of mechanisms, not just due to structural changes in the Spike protein. Are there plans underway for structural elucidation of other proteins/complexes apart from Spike?
3. The variants that have posed most health complications in the South East Asian countries could be mentioned.
4. HLA repertoire differences can occur not only because of being in close contact with animals but also because of the different climatic/ environmental conditions. What are the major differences in the HLA repertoires of the individuals in South East Asian countries in comparison with others?
5. Can healthy individuals who have been affected by after-effects of the vaccine also be included in the cohort?
6. The SEA consortium must network well with neighboring SA and other countries, as once any advantageous mutations are gained, the variant will spread rapidly and become the dominant form.
7. While statistical validation is a very intrinsic and necessary part of the whole endeavor, completely new integrative ways of looking at and analyzing the data will be required in view of the diverse data types gathered from this kind of a project.
8. A focused effort will be required to frame key biological questions and to collect the key parameters related to variant biology, clinical outcomes, long-Covid and breakthrough infections. Once such information is available, it would be beneficial to harness AI/ML techniques.  
9. While all the named components of the project are very important, the computational genomics has not been sufficiently outlined in my opinion.
10. I wish the investigators and all involved a brilliant scientific journey ahead.

Does the article adequately reference differing views and opinions?

Yes

Are all factual statements correct, and are statements and arguments made adequately supported by citations?

Yes

Is the Open Letter written in accessible language?

Yes

Where applicable, are recommendations and next steps explained clearly for others to follow?

Yes

Is the rationale for the Open Letter provided in sufficient detail?

Yes

Reviewer Expertise:

SARS CoV-2, Structural Biology, Computational Biology, Infectious disease, Cardiovascular disease

We confirm that we have read this submission and believe that we have an appropriate level of expertise to confirm that it is of an acceptable scientific standard, however we have significant reservations, as outlined above.
